# Enhanced Genetic Tools for Engineering Multigene Traits into Green Algae

**DOI:** 10.1371/journal.pone.0094028

**Published:** 2014-04-07

**Authors:** Beth A. Rasala, Syh-Shiuan Chao, Matthew Pier, Daniel J. Barrera, Stephen P. Mayfield

**Affiliations:** California Center for Algae Biotechnology and Division of Biological Sciences, University of California San Diego, La Jolla, California, United States of America; Nanyang Technological University, Singapore

## Abstract

Transgenic microalgae have the potential to impact many diverse biotechnological industries including energy, human and animal nutrition, pharmaceuticals, health and beauty, and specialty chemicals. However, major obstacles to sophisticated genetic and metabolic engineering in algae have been the lack of well-characterized transformation vectors to direct engineered gene products to specific subcellular locations, and the inability to robustly express multiple nuclear-encoded transgenes within a single cell. Here we validate a set of genetic tools that enable protein targeting to distinct subcellular locations, and present two complementary methods for multigene engineering in the eukaryotic green microalga *Chlamydomonas reinhardtii*. The tools described here will enable advanced metabolic and genetic engineering to promote microalgae biotechnology and product commercialization.

## Introduction

Microalgae have recently attracted attention as potential low-cost platform for the production of a broad range of commercial products including biofuels, nutraceuticals, therapeutics, industrial chemicals and animal feeds [1]–[11]; and genome engineering will enable and enhance algae-produced bio-products [1], [5], [6], [12]–[19]. However, while much has been written about the potential of transgenic microalgae, little of that potential has yet to be commercialized. A major obstacle to generating useful transgenic algae strains has been the lack of molecular tools and overall poor expression of heterologous genes from the nuclear genome of many microalgae species, at least partially due to rapid gene silencing [20]–[23]. For example, a set of validated vectors for targeting transgene products to specific subcellular locations do not exist, nor does the vector to allow the expression of multiple nuclear-encoded genes within a single cell.

Previously, we described a nuclear expression strategy that overcomes transgene silencing by using the foot-and-mouth-disease-virus (FMDV) 2A “self-cleaving” peptide to transcriptionally fuse transgene expression to the antibiotic resistance gene *ble* in the green microalga *Chlamydomonas reinhardtii* [23], [24]. It is believed that the FMDV 2A sequence “self-cleaves” through ribosome-skipping during translation rather than a proteolytic reaction, and has been termed CHYSEL (*cis*-acting hydrolase element) [25], [26]. This strategy allowed for the selection of transgenic lines that efficiently express the transgene-of-interest, and this robust expression remains for many generations. We demonstrated the utility of our pBle-2A vector with the expression and secretion of the valuable industrial enzyme, xylanase [23]. Furthermore, this expression strategy enabled, for the first time, the robust expression of six fluorescent proteins (FPs) in the cytosol of green microalgae [24]. FPs have become essential research tools that have revolutionized many fields of biology

Here we report the construction and validation of a set of transformation vectors that enable protein targeting to distinct subcellular locations, and present two complementary methods for multigene engineering in the eukaryotic green microalga *C. reinhardtii*.

## Results

**Figure 1:**
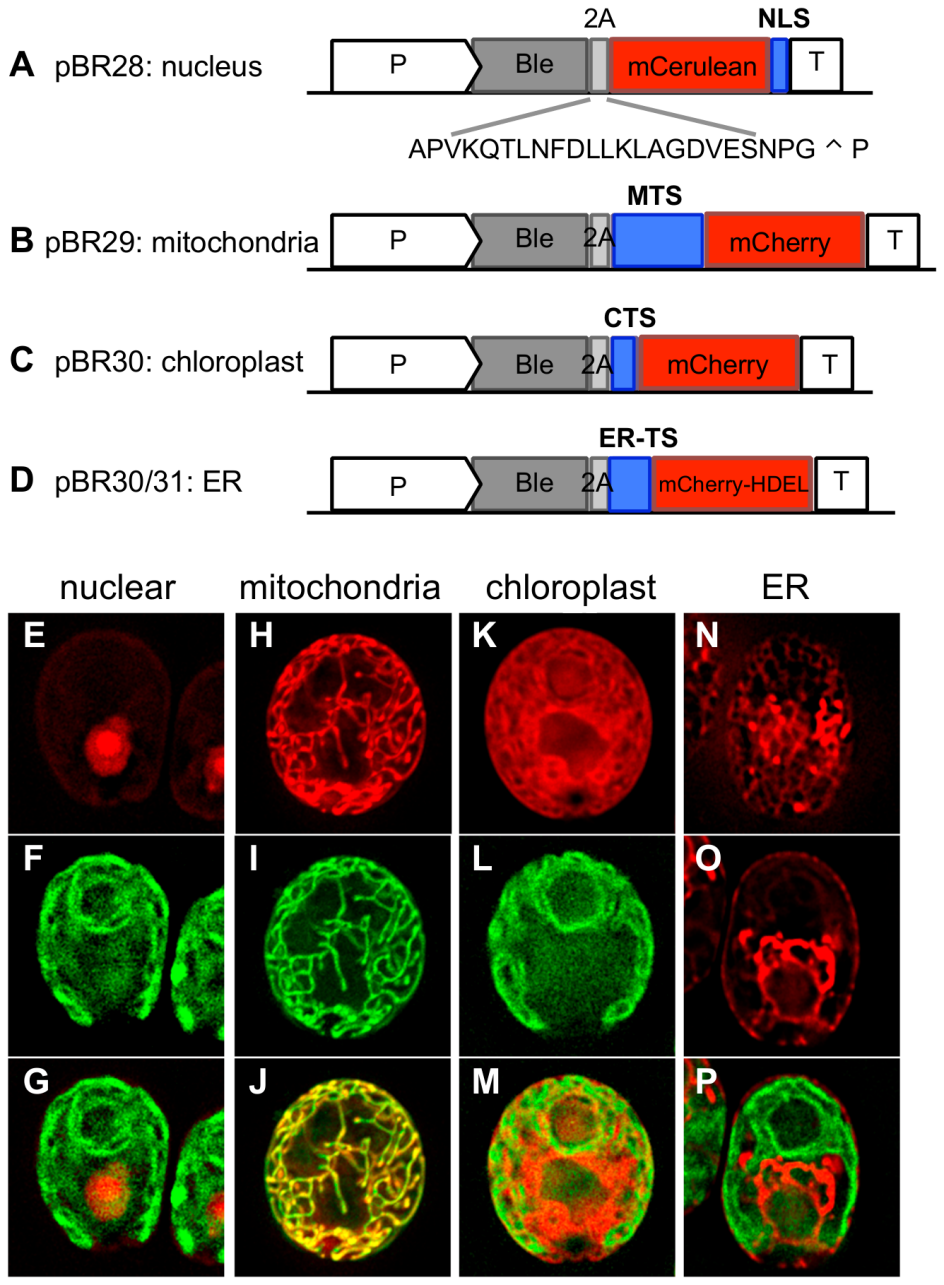
*Chlamydomonas* transformation vectors for protein targeting to specific subcellular locations. A–D. Schematic representation of *Chlamydomonas* targeting vectors. All transformation vectors contain the hsp70/rbcs2 promoter (P), the *ble* gene that confers resistance to zeocin, the 2A self-cleaving sequence from foot-and-mouth-disease virus, and the rbcs2 terminator (T). The site of cleavage is indicated with an arrowhead. A. pBR28, mCerulean is targeted to the nucleus by a C-terminal fusion to 2xSV40 NLS. B. pBR29, mCherry is targeted to the mitochondria by an N-terminal fusion to the mitochondrial transit sequence (MTS) of mitochondrial atpA. C. pBR32, mCherry is targeted to the chloroplast using the chloroplast transit sequence (CTS) from psaD. D. pBR30/31, mCherry is targeted to the ER using the ER-transit sequence (ER-TS) from either BiP1 or ars1. The ER retention sequence H-D-E-L is fused to the C-terminus of mCherry. E–P. Microscopy images of cells transformed with pBR28 (E–G), pBR29 (H–J), pBR32 (K–M), and pBR30 (N–P). Top row are live cell images of the fluorescent proteins targeted to the nucleus (E), mitochondria (H), chloroplast (K) or ER (N, O). (I) The cell is co-stained with the mitochondrial dye Mitotracker. (N) Cross section through the top of a cell expressing mCherry in the ER allows for the visualization of the cortical ER network. (O) Cross section through the middle of the same cell as in (N). The chloroplast membranes are visualized in (F), (L) and (P). Merged images are shown in the bottom row.

Here we describe vectors that enable protein targeting to four important organelles: the nucleus, mitochondria, endoplasmic reticulum (ER), and chloroplast (Table 1). The nucleus houses the majority of the cell's genetic material, and therefore is critical for the regulation of most gene expression. To generate a nucleus-targeting vector, a tandem copy of the nuclear localization signal (NLS) from simian virus 40 (SV40) [27] was fused to the C-terminus of mCerulean, and transcriptionally linked to ble-2A (Figure 1A). Cells transformed with mCerulean-2xNLS displayed fluorescence signals that were concentrated in the nucleus (Figure 1E-G, Figure S1A), as confirmed by co-staining fixed mCerulean fluorescent cells with the nuclear DNA-stain Hoechst (Figure S1B).

**Table 1:** Summary of transit peptides and targeting sequences used in this study.

Vector	Location	Transit sequence	Size	Function	Reference
pBR28	Nuclear	2x SV40 NLS	20 aa	Tandem copy of a nuclear localization sequence from SV40	commonly used in mammalian vectors
pBR29	Mitochondria	atpA	45aa	Alpha subunit of mitochondrial ATP synthase.	this report
pBR30	ER	BIP1	31aa	Chaperone, Hsp70 superfamily.	this report
pBR31	ER	ARS1	30aa	Periplasmic protein involved in mineralization of sulfate by hydrolyzing sulfate esters.	Rasala et al., 2012
pBR32	Chloroplast	PSAD	35aa	Protein of Photosystem I.	Fischer and Rochaix, 2001

Mitochondria function in respiration, producing ATP via oxidative respiration, and therefore play an essential role in cell metabolism. Mitochondria also function in the metabolism of amino acids, lipids, iron, calcium homeostasis, apoptosis and cell signaling. To generate a mitochondria-targeting vector, the N-terminal mitochondria transit sequence (MTS) from the nuclear gene encoding the alpha subunit of the mitochondrial ATP synthase located in the mitochondrial matrix, was fused between ble-2A and mCherry (Figure 1B). Live cell microscopy of independent clones transformed with MTS-mCherry shows mCherry signal localized to tubular mitochondrial networks [28]–[30] (Figure 1H, Figure S1D), which was confirmed by co-localization with Mitotracker, a mitochondria-specific dye (Figure 1I, J, Figure S1D).

The most studied *C. reinhardtii* organelle is the chloroplast, the site of photosynthesis. *C. reinhardtii* has a single cup-shaped chloroplast that occupies about 75% of the volume of the cell. The chloroplast is also the site of multiple metabolic reactions, including the biosynthesis of amino acids, isoprenoids, fatty acids, and starch [31]. The chloroplast transit sequence (CTS) from the photosystem I protein psaD was chosen for the chloroplast-targeting vector (Figure 1C), as it has been used previously to target heterologous proteins to the chloroplast in *C. reinhardtii* [32]. Cells transformed with the ble2A-CTS-mCherry vector displayed red fluorescence signals that properly localized to the chloroplast (Figure 1K, Figure S1E) and partially overlapped with chloroplast auto-fluorescence derived from chlorophyll and other pigments localized in chloroplast photosynthetic membranes (Figure 1L, M)

The endoplasmic reticulum (ER) forms an extensive interconnected network of tubules and flattened stacks located throughout the cytoplasm and is continuous with the nuclear envelope [33]. The ER has multiple functions, including translocation and modification of proteins destined for secretion. It also functions in lipid metabolism, carbohydrate metabolism, and detoxification. Two ER-targeting vectors were created by fusing the ER signal sequence of the *C. reinhardtii* genes *ars1* or *bip1* between ble-2A and mCherry; and the ER retention signal His-Asp-Glu-Leu (HDEL) [34] was fused to the C-terminus of mCherry (Figure 1D). *ars1* encodes for a secreted arylsulfatase [35], and its signal peptide has been shown to target heterologous xylanase 1 for secretion [23]. BiP1 is an ER-localized chaperone of the HSP70 superfamily [36]. Using live cell microscopy, cells transformed with either ER-targeting vector displayed mCherry localization to reticular, net-like structures under the plasma membrane, which are reminiscent of cortical ER that has been characterized in other eukaryotes (Figure 1N). Z planes focused through the middle of cells demonstrate that mCherry localizes to a structure that is continuous with the nuclear envelope (Figure 1O, P, Figure S1C). While we were unable to identify any ER-specific dyes that function in *Chlamydomonas*, we are confident that both ars1-mCherry-HDEL and BiP1-mCherry-HDEL successfully targets the FP to the ER, based on the resultant distinct and characteristic localization pattern.

To verify the fluorescence live cell microscopy data, SDS-PAGE immunoblotting was performed on lysates from individual transformants expressing the targeting vectors described above. Immunoblots demonstrate that the targeted fluorescent proteins accumulate to detectible levels, are correctly processed from ble-2A, and display the predicted mobility for the respective mature protein (Figure S2).

**Figure 2:**
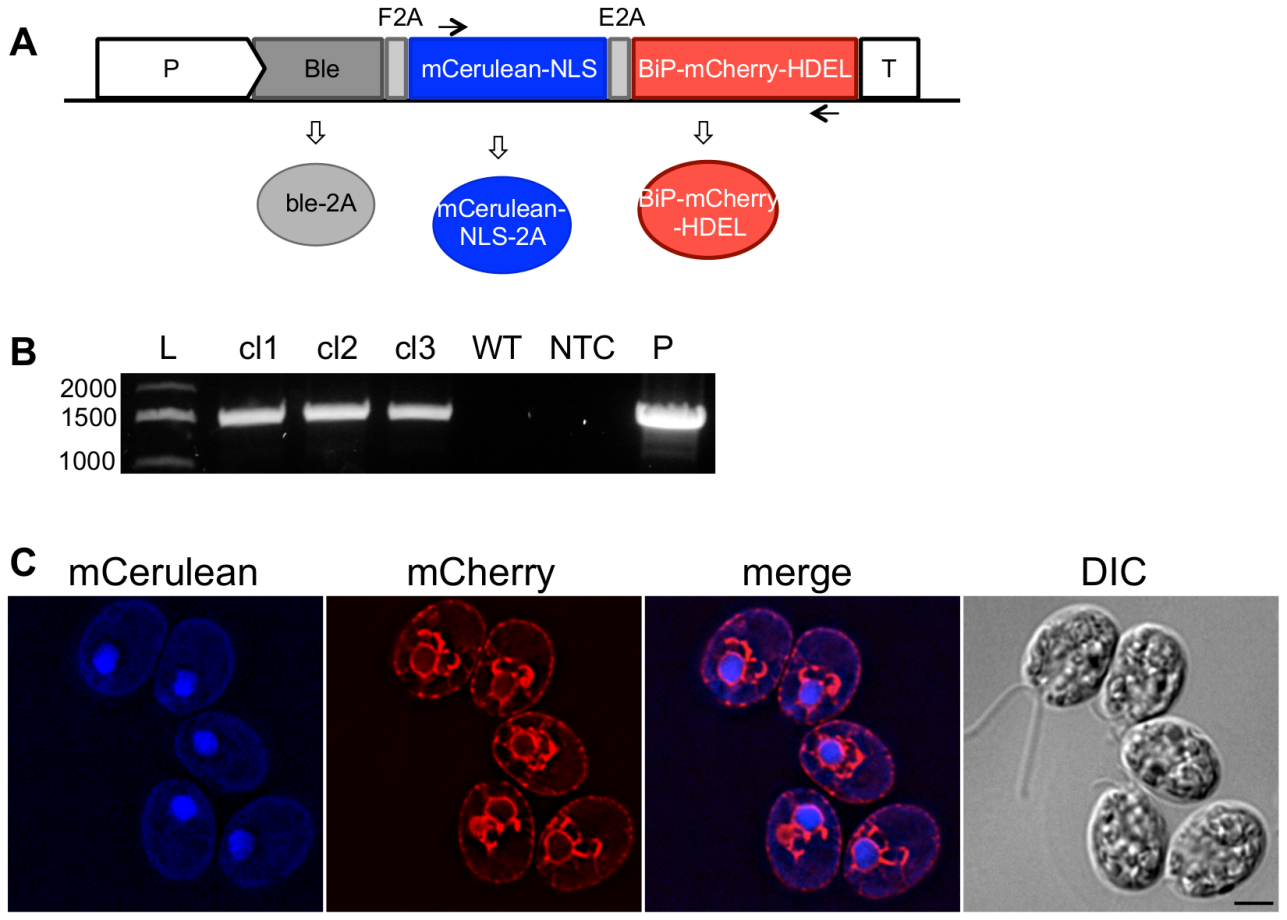
Gene stacking using a multi-cistronic transformation vector. A. A schematic representation of the *Chlamydomonas* multi-cistronic expression vector. The expression of the cassette is under the control of the hsp70/rbcs2 promoter (P). *Ble* confers zeocin-resistance. mCerulean is targeted to the nucleus with the SV40-NLS. mCherry is targeted to the ER using the BiP ER-TS and the HDEL retention sequence. Two 2A self-cleaving sequences are fused between the three cistrons: F2A, from FMDV1; E2A, from equine rhinitis A virus. Black arrows represent the location of the oligonucleotides used in (B). Following co-translational processing of the 2A peptides, three distinct proteins are expressed (ovals). B. PCR analysis of the multi-cistron cassette genome integration. Transformants were screened by PCR to identify individual clones that correctly integrated the multi-cistronic transformation vector, using the oligonucleotides indicated by the arrows in (A). Three independent clones (cl) are shown. L, ladder; WT, wildtype cc1690; NTC, no template control; P, plasmid. C. Live cell fluorescence microscopy of a clone expressing the multi-cistronic vector. mCerulean-NLS (blue) localizes to the nucleus while BiP-mCherry-HDEL (red) is targeted to the ER. Scale bar, 5 μm.

Several successful strategies for the coordinated expression of multiple genes have been described in other eukaryotes. These include the use of FMDV-2A and 2A-like peptides to ensure transcriptional co-expression of multiple proteins encoded in a single open reading frame (ORF) with co-translational “cleavage” into distinct peptides [26]. However, with the exception of a gene-of-interest and an antibiotic resistance marker, coordinated multi-gene expression has yet to be achieved in green microalgae. We modified our ble2A expression strategy to include a second 2A peptide from equine rhinitis A virus (E2A) [37] followed by a third protein coding sequence (Figure 2A). Transgenic algae expressing the Ble•E2A−mCerulean•2xNLS•F2A−BiP•mCherry•HDEL ORF were recovered which had properly integrated the multi-cistron transgene cassette and accumulated mCerulean in the nucleus and mCherry in the ER (Figure 2B, C), both at high levels of expression.

**Figure 3:**
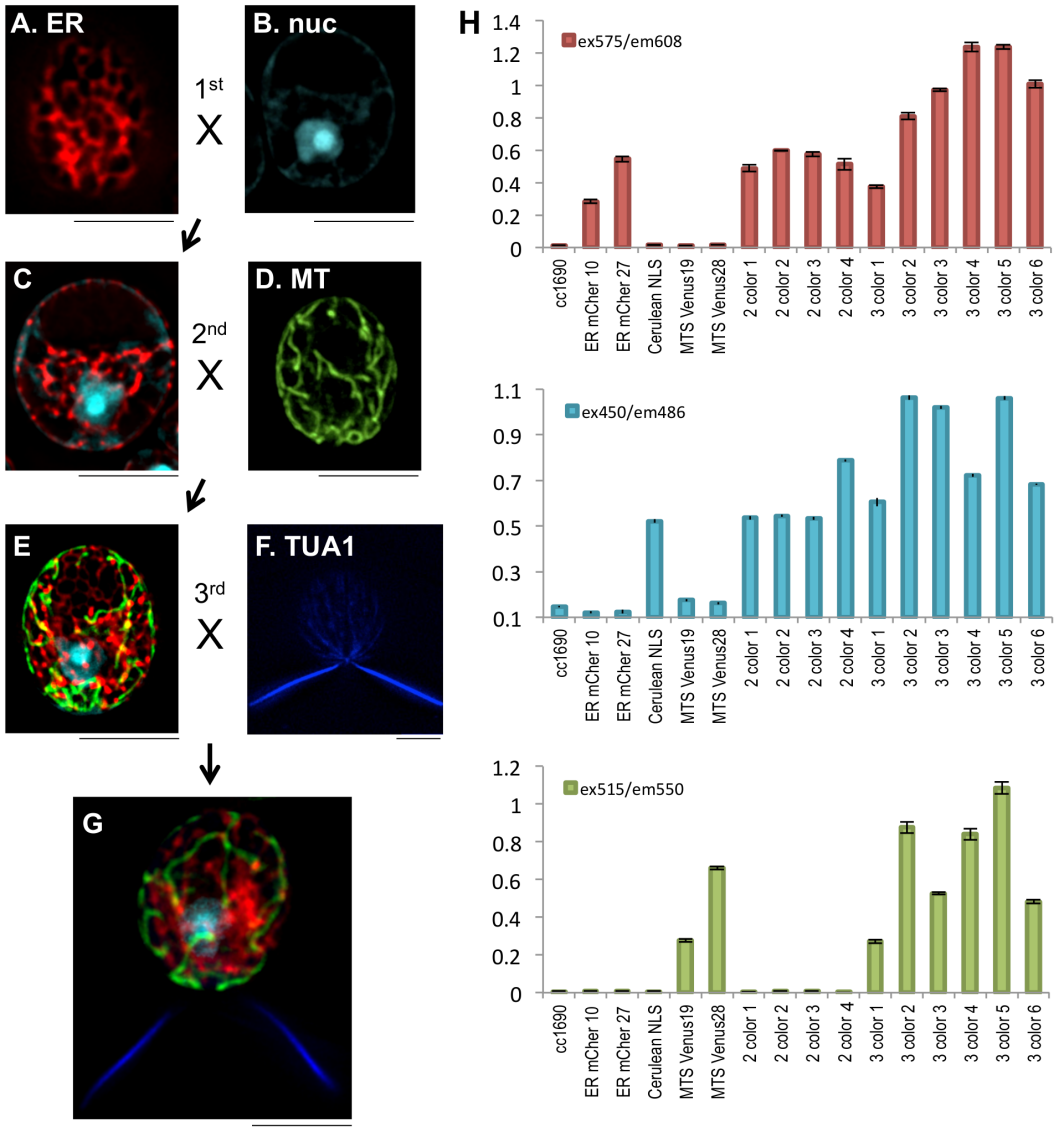
Gene stacking through mating. An mt+ strain transformed with pBR30 (A) was crossed with an mt- strain transformed with pBR28 (B). Progeny that expressed mCherry in the ER and mCerulean in the nucleus were obtained (C). Cell lines expressing both ER-mCherry and nuclear mCerulean were crossed with cells transformed with mitochondria-targeted Venus (D), to obtain progeny that stably expressed three distinct FPs in three sub-cellular locations (E). These cell lines were crossed to transgenic cells that expressed α-tubulin (TUA1) fused to mTagBFP (F), to obtain progeny that expressed four FPs in four distinct subcellular locations (G). H. Fluorescence plate reader assays of the parents and progeny indicate that FP expression remains stable following matings. Cell lines were assayed for mCherry expression (ex575/em608), mCerulean expression (ex450/em486), and Venus expression (ex515/em550). ER-mCherry parents (ER-mCher 10 and 27), nuclear mCerulean parent (Cerulean NLS) and mitochondrial Venus parents (MTS Venus 19 and 28) are shown along with WT cc1690. 2 color cell lines 1–4 express ER-mCherry and Cerulean-NLS. 3 color cell lines 1–6 express ER-mCherry, nuclear mCerulean and mitochondrial Venus. Scale bars, 5 μm.

A potential disadvantage of the double 2A vector is that the 2A C-terminal fusion to the middle protein of the poly-cistron may disrupt its function and/or localization. Thus, we developed a gene-stacking strategy to generate transgenic algae that express up to four targeted proteins by harnessing the power of genetic breeding. *C. reinhardtii* is a haploid organism that normally divides vegetatively. However, under certain conditions, a mating-type plus (mt+) gamete will mate with a mating-type minus (mt-) gamete to form a diploid zygospore that then undergoes meiosis to yield four haploid progeny. During this mating, genes integrated into separate chromosomes can individually assort resulting in progeny with genes from either parent. To test whether we could cross two transgenic lines and obtain a single progeny that contained both transgenes that were still expressed at desirable levels, we mated an mt+ strain that expressed mCherry targeted to the ER (Figure 3A) to an mt- strain that expressed mCerulean targeted to the nucleus (Figure 3B), both as ble2A fusions. The progeny were FACS sorted for cells that expressed both mCerulean and mCherry. The presence and expression of both inherited transgenes was verified by PCR analysis and fluorescence microplate reader analysis (Figure 3H and data not shown). Live cell microscopy confirmed that both engineered genes from the transgenic parents, nuclear-localized mCerulean and ER-localized mCherry, were inherited in selected progeny (Figure 3C). This process was repeated, mating the two-colored algae to an additional transgenic line expressing mitochondria-targeted Venus (Figure 3D) to obtain progeny that robustly expressed three engineered transgenes within a single cell (Figure 3E). After a third round of mating between the 3-colored strain with a strain that stably express alpha-tubulin fused to mTagBFP (Figure 3F), we obtained progeny that expressed four different FPs, all properly localized to four distinct subcellular locations: the nucleus, ER, mitochondria, and flagella (Figure 3G).

To determine whether the two gene stacking approaches described above could be used in combination, we mated three independent clones that stably express proteins from the multi-cistron double-2A vector, to three independent clones of the opposite mating type that express β-glucuronidase (GUS) marked with hygromycin-resistance. GUS is an enzyme involved in the catalysis of carbohydrates and is widely used as a reporter. Progeny from the cross were selected on TAP agar plates containing both zeocin and hygromycin B, and screened for the presence of the Ble•E2A−mCerulean•2xNLS•F2A−BiP•mCherry•HDEL ORF by PCR as described above. Progeny from 8 of the 9 matings retained the multi-cistron ORF in greater than 85% of the progeny screened (Figure S3A), suggesting that the large expression cassette does not undergo rearrangement during meiosis. Importantly, the majority of the progeny express mCherry and mCerulean to the same or even slightly better levels than the parents (Figure S3C, D). The progeny were also screened for the presence and expression of the GUS gene. As expected, the majority of the hygromycin-resistant progeny also retained the genetically linked GUS gene (Figure S3B). Interesting, while the parents displayed poor expression of β-glucuronidase likely due to transgene silencing, most of the progeny tested displayed significant GUS enzyme activity (Figure S3E).

## Discussion

The use of transgenic microalgae for the production of bioproducts has enormous economic and biotechnology promise, because algal production combines the simplicity and speed of haploid, single-cell genetics in an organism with elaborate biosynthetic potential, and with the associated economic benefit of using photosynthesis to drive product formation. Here we describe key genetic tools that will enable complex genetic and metabolic engineering in green microalgae: the ability to target gene products to specific subcellular locations, and vectors and well-characterized protocols that enable multi-gene stacking within a single transgenic cell. We have generated a class of nuclear transformation vectors that efficiently and specifically target transgene products to the nucleus, mitochondria, ER and chloroplast (Figure 1). Furthermore, the transit sequences can be used in combination with multiple 2A self-cleaving sequences to generate multi-cistron vectors that enable robust and coordinated expression of multiple recombinant proteins from a single transcript (Figure 2). Finally, we describe methods to stack up to four transgenes within a single cell (Figure 3 and Figure S3). Importantly, transgene expression remains robust throughout the mating process, suggesting that the microalgal silencing mechanism(s) are not activated during gametogenesis or meiosis.

Sophisticated genetic engineering often requires the coordinated expression of more than one gene. For example, multi-gene engineering has been used for therapeutics [38]–[40], and metabolic engineering [41]–[43]. Metabolic networks are complex in all eukaryotic organisms including algae, and individual biochemical steps of a single pathway can sometimes take place in multiple subcellular compartments [44], [45]. Thus, in order to achieve complex genetic and metabolic engineering in microalgae, transformation vectors that target multiple proteins (enzymes) to specific cellular locations – such as the ones described above - are required.

One of the biggest challenges to nuclear genome engineering in *C. reinhardtii* is transgene silencing [20]–[23]. For example, when mCerulean or GUS are directly linked to the P_AR1_ promoter and integrated into the nuclear genome, the reporter proteins are nearly undetectable (Figure S4). Previously, we developed an expression strategy to overcome transgene silencing by transcriptionally linking the transgene-of-interest to the selection marker Ble through a 2A self-cleaving sequence [23]. Here, we demonstrate that this expression strategy can be used in combination with organelle targeting sequences to direct protein localization to desired sub-cellular locations. Furthermore, our data show that the targeted proteins are well-expressed. Ble is the most effective selection marker for overcoming silencing tested thus far (our unpublished data). This is likely because Ble functions by sequestration rather than enzymatic inactivation, binding to zeocin in a 1-to-1 ratio [46]. Thus high levels of Ble expression are required to survive zeocin selection. However, the ability to use only one selection marker limits the utility of the ble-2A expression strategy. The double 2A multi-cistron vector was developed to overcome this limitation. Indeed, the multi-cistron vector was used to co-express two reporter proteins that were directed to two distinct subcellular locations. We further demonstrate that the double 2A vector is stable; the similar 2A sequences do not recombine during vegetative cell division or meiosis to loop out the middle coding sequence.

A second gene-stacking strategy investigated was gene-stacking though mating. Two strains of opposite mating types engineered to express ble-2A-ER-mCherry or ble-2A-mCerulean-NLS were mated, germinated on TAP/zeocin plates and then FACS sorted for mCherry and mCerulean. Even though progeny were selected on zeocin, either ble2A construct could provide antibiotic resistance. Thus, there was only selection pressure for the expression of one – and not both – of the ble2A constructs. Notably, however, we were able to recover strains that robustly expressed both mCherry and mCerulean. This result was repeated with three and then four transgenes. Indeed, even when we were unable to distinguish mTagBFP from mCerulean by FACS and therefore progeny could not be enriched by flow cytometry, we were still able to easily recover strains in which mCerulean-NLS and mTagBFP-TUA were well expressed. These data suggest that the robust silencing mechanisms that are well-described but poorly understood may not affect transgenes once they have escaped the initial mechanism of silencing upon transformation and integration.

Indeed, our data indicate that transgene silencing may even be lessened following mating and meiosis. Two independent transgenic strains expressing a silenced *GUS* gene were mated and *GUS*-positive progeny were assayed for GUS expression (Figure S3). Most of the progeny from both crosses displayed significantly more GUS activity than the parent strains. We are currently investigating the molecular mechanism behind these notable results.

Microalgae are poised to revolutionize many industries including energy, nutrition, health, and specialty chemicals. The molecular genetic tools and methods described here for multi-gene engineering and protein targeting will significantly advance the current state of microalgae genetic, metabolic, and pathway engineering, and therefore impact the development of transgenic algae as a biotechnology platform.

## Material and Methods

### Algal strains, transformations and growth conditions

The *C. reinhardtii* strains used in this study were cc1690 (mt+) and cc1691 (mt-, Chlamydomonas Resource Center). Cells were transformed by electroporation as described previously [23]. Transformants were selected on TAP (Tris–acetate–phosphate) agar plates supplemented with 2.5–10 μg/ml zeocin. Transformants were screened by PCR to identify gene positive transformants as described previously [23].

### Plasmid construction

mCherry, Venus, mCerulean, and mTagBFP were codon-optimized for expression from the nuclear genome of *C. reinhardtii*, as previously described [24]. The organelle transit sequences from *bip1*, *psaD*, and *atpA* were PCR-amplified from genomic DNA isolated from cc1690, using the oligonucleotides described in Table S1, and fused between ble2A and mCherry in the pBR9 vector [24] using the GeneArt Seamless Cloning Kit (Life Technologies, Carlsbad, CA). The 2x SV40 NLS was codon-optimized for *C. reinhardtii* nuclear expression, synthesized as sense and antisense single stranded oligonucleotides, annealed, and cloned into pBR25 [24] that had been digested with BamHI and EcoRI. His-Asp-Glu-Lys (HDEL) was fused to the end of mCherry by PCR using a reverse oligonucleotide encoding for the ER retention sequence. mCherry-HDEL was cloned behind Ble-2A-BiP or Ble-2A-ARS1 [23] by restriction digest and ligation using the enzymes XhoI and BamHI. To generate pBR26 double-2A vector, the 2A sequence from equine rhinitis A virus (E2A) [37] was first codon-optimized, synthesized, and tested for self-cleavage in *C. reinhardtii* (our unpublished data). pBR30 was linearized by PCR and mCerulean-NLS and E2A were fused between Ble2A and BiP-mCherry-HDEL using the GeneArt Seamless Plus Cloning Kit (Life Technologies). GUS was codon-optimized for expression from the nuclear genome, synthesized and cloned into the pBR2 hygromycin resistance expression cassette [23] by restriction digest with NdeI and BamHI.

### Fluorescence microscopy

Representative clones were grown in TAP media without antibiotics to late log phase on a rotary shaker. Mitotracker Green FM (Life Technologies) was used to stain the mitochondria of live cells as per the manufacturer's instructions. Live cells were plated on TAP/1% agar pads prior to image acquisition. Images were captured on a Delta Vision (Applied Precision Inc., Issaquah, WA) optical sectioning microscope system composed of an Olympus IX71 inverted microscope (Center Valley, PA) equipped with an Olympus UPlanSApo 100×/1.40 objective and a CoolSNAP HQ2/ICX285 camera (Photometrics, Tucson, AZ). The following filters were used: mTagpBFP, excitation 360/40 nm, emission 457/50 nm; mCerulean, excitation 436/10 nm, emission 470/30 nm; Venus, excitation 470/40 nm, emission 515/30 nm; mCherry, excitation 558/28 nm, emission 617/73 nm; and Mitotracker Green FM, excitation 470/40 nm, emission 515/30 nm. Image acquisition and deconvolution were performed using Resolve3D SoftWoRx-Acquire (Version 5.5.1, Applied Precision Inc). Brightness and contrast were adjusted using Adobe Photoshop CS3 or ImageJ software. The images in Figure S1 were adjusted identically.

### Fluorescence microplate reader assay

Cells were grown in TAP media without antibiotics until late log phase. 100 μls of cells were transferred, in triplicate, to wells of a black 96 well plate (Corning Costar, Tewksbury MA), and fluorescence was read using a Tecan plate reader (Tecan Infinite M200 PRO, Männedorf, Switzerland). Fluorescence readings with the indicated excitation/emission filters were acquired using a calculated optimal gain, which was determined prior to each reading. TAP media was used to blank the readings. Fluorescence signals were normalized by chlorophyll fluorescence (excitation 440/9 nm, emission 680/20 nm).

### Chlamydomonas matings

Matings were performed using the following protocol: gametes were generated by incubating mt+ and mt- cells overnight in nitrogen-free liquid TAP. Mt+ gametes were mixed with mt- gametes for 2–4 hours, and the mating reactions were plated to TAP/3% agar plates and incubated in the dark for 5–7 days. Unmated cells were scraped off to the side using a sterile razor blade and the plates were subjected to chloroform treatment to kill any remaining unmated cells. For the matings described in Figure 3, spores were collected using an inoculating loop and struck onto TAP agar plates supplemented with 10 μg/ml of zeocin and incubated in the light until colonies appeared. For the matings described in Figure S3, spores were inoculated into 50 ml TAP and grown in light until late log phase. Cells were then plated to TAP agar plates containing 10 μg/ml of zeocin and 15 μg/ml of hygromycin B.

### Fluorescence activated cell sorting

Progeny from matings were inoculated into TAP liquid cultures without antibiotics and grown to late log phase, diluted back 1∶10, and grown for another 12–24 hours. The cultures were then sorted for expression of the appropriate fluorescence proteins on a BD Influx cell sorter (BD Biosciences, Vannas, Sweden), gating for mCherry+ and mCerulean+ for the 2-color strain; and mCherry+, mCerulean+ and Venus+ for the 3- and 4-color strain. Sorted progeny were verified by PCR analysis to confirm the presence of the targeted-FP genes, and by plate reader assay prior to microscopy. mCherry-expressing cells were identified using a 532 nm laser and a 585/40 nm filter to detect red fluorescence. mCerulean-expressing cells were identified using a 457 nm laser and a 480/40 nm filter to detect cyan fluorescence. Venus-expressing cells were identified using a 488 nm laser for excitation and a 530/40 nm filter. mTagBFP fluorescence could not be distinguished from mCerulean fluorescence in the 4-color strain, rather cells were sorted for cyan/blue, yellow, and red fluorescence and then verified by PCR analysis and plate reader assay for the presence of all four desired FP genes.

### GUS activity assay

Cell cultures were grown in liquid TAP media without antibiotics to late log phase in 12-well plates. 100 μls of cells were incubated with 25 μls of 1 mg/ml 4-methylumbelliferyl-beta-D-glucuronide (Sigma, St. Louis, MO) in a 96-well black microplate for 20 minutes. GUS activity was determined using a fluorometric assay, by measuring the accumulation of the fluorescent product (ex365/em455).

## Supporting Information

Figure S1 Fluorescence microscopy of *Chlamydomonas* cells transformed with the targeting vector. Transgenic lines are compared with wildtype cc1690 using fluorescence microscopy. Images were acquired and adjusted identically for each group. Chl, chloroplast auto-fluorescence. A. Live cell microscopy of three independent cell lines transformed with pBR28, nucleus-targeted mCerulean. Top row, mCerulean; middle row, chloroplast autofluorescence; third row, merge; and fourth row, differential interference contrast microscope images. Images from cc1690 are shown for comparison and were acquired and adjusted identically. B. Fluorescence microscopy on fixed cells expressing mCerulean-NLS, and stained with Hoechst. Hoechst stains DNA and was used as a marker for the nucleus. C. Live cell fluorescence microscopy on cell lines expressing pBR30 or pBR31, ER-targeted mCherry. Z-sections of cells expressing ER-targeted Cherry: top row, focal plane through the top of the cells revealing the cortical ER; second row, focal plane through the middle of the cells, revealing mCherry localization to the ER that is continuous with the nuclear envelope. D. Live cell microscopy of three independent cell lines transformed with pBR29, mitochondria-targeted mCherry (red). Cells are co-stained with mitochondrial dye Mitotracker (green). E. Live cell microscopy of a cell line transformed with pBR32, chloroplast-targeted mCherry (red). Non-targeted mCherry, which accumulates in the cytoplasm and nucleus, is shown for comparison (cyt). Scale bars, 5 μm.
(TIF)

Figure S2 Targeted fluorescent proteins are well-expressed. Immunoblot analysis of total soluble protein from clones transformed with cytoplasmic (cyt) mCerulean (A) or mCherry (B, C) compared to mCerulean targeted to the nucleus (A), mCherry targeted to the chloroplast (B), or mCherry directed to the ER and mitochondria (C). Molecular weight size markers, 31 kDa and 24 kDa, are shown for each gel. Mobility shifts in (A) reflect the addition of the 2xNLS nuclear localization sequence (2.3 kDa). Slight mobility shifts in (B) reflect the addition of the HDEL ER retention sequence (0.5 kDa). Immunoblots probed for atpB are shown as loading controls.
(TIF)

Figure S3 Combining gene-stacking approaches: mating strains transformed with the multicistron vector. Three independent clones stably transformed with pBR26 were mated, pairwise to 3 independent clones expressing β-glucuronidase (GUS). A) A PCR screen with primers shown in Figure 2a on 46 progeny from two representative matings: pBR26 cl57 x pBR2 GUS cl55 (lanes A–D) and pBR26 cl4 x pBR2 GUS cl45 (lanes E–H). (-) cc1690 lysate; (+) pBR26 plasmid. A band indicates the stable inheritance of the multicistron expression cassette from parent to progeny. B) The lysates in (A) were screened with primers specific to GUS. C) Fluorescence microplate reader assay detecting mCherry signals in 12 progeny per mating for the two representative matings shown in a. D) Fluorescence microplate reader assay detecting mCerulean signals in 12 progeny per mating for the two representative matings shown in A. C and D) Y-axis is the ratio of relative fluorescence units of the fluorescent protein to that of chlorophyll fluorescence (ex440/em680). E. Fluorometric GUS activity assay. RFU, relative fluorescence units, reflects the amount of GUS substrate that is catabolized.
(TIF)

Figure S4 Transgenes expressed from the nuclear genome of C. reinhardtii are subjected to gene silencing. A. Fluorescence plate reader assay on individual transgenic clones expressing P_AR1_:mCerulean is compared to a clone that is transformed with P_AR1_:ble2A-mCerulean. The Y-axis represents normalized fluorescence, which is mCerulean fluorescence (ex 450/em486) divided by chlorophyll fluorescence (ex440/em680). B. β-glucuronidase activity assays were performed on individual clones transformed with P_AR1_:GUS compared to a clone expressing P_AR1_:ble2A-GUS. GUS activity was monitored by accumulation of fluorescent product. Relative fluorescent units is shown on the Y-axis.
(TIF)

Table S1 Oligonucleotides used in this study to construct Chlamydomonas targeting vectors.
(TIF)
