# Supplementary figures and images for: Enhanced Genetic Tools for Engineering Multigene Traits into Green Algae
Source: PLoS One. 2014 Apr 7;9(4):e94028. doi: 10.1371/journal.pone.0094028 (PMC3978050; doi:10.1371/journal.pone.0094028)

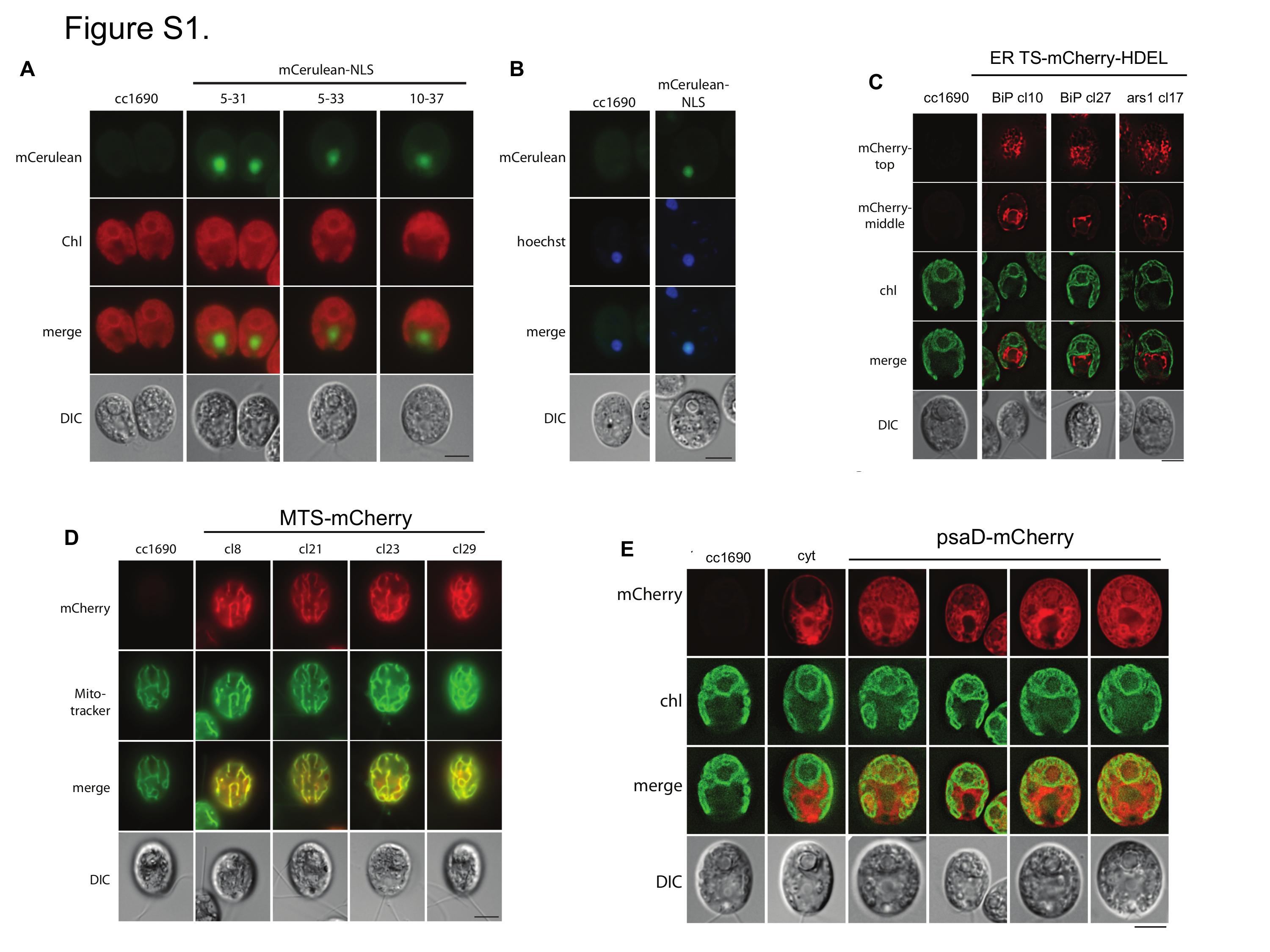

Supplement: Figure S1 — Fluorescence microscopy of Chlamydomonas cells transformed with the targeting vector. Transgenic lines are compared with wildtype cc1690 using fluorescence microscopy. Images were acquired and adjusted identically for each group. Chl, chloroplast auto-fluorescence. A. Live cell microscopy of three independent cell lines transformed with pBR28, nucleus-targeted mCerulean. Top row, mCerulean; middle row, chloroplast autofluorescence; third row, merge; and fourth row, differential interference contrast microscope images. Images from cc1690 are shown for comparison and were acquired and adjusted identically. B. Fluorescence microscopy on fixed cells expressing mCerulean-NLS, and stained with Hoechst. Hoechst stains DNA and was used as a marker for the nucleus. C. Live cell fluorescence microscopy on cell lines expressing pBR30 or pBR31, ER-targeted mCherry. Z-sections of cells expressing ER-targeted Cherry: top row, focal plane through the top of the cells revealing the cortical ER; second row, focal plane through the middle of the cells, revealing mCherry localization to the ER that is continuous with the nuclear envelope. D. Live cell microscopy of three independent cell lines transformed with pBR29, mitochondria-targeted mCherry (red). Cells are co-stained with mitochondrial dye Mitotracker (green). E. Live cell microscopy of a cell line transformed with pBR32, chloroplast-targeted mCherry (red). Non-targeted mCherry, which accumulates in the cytoplasm and nucleus, is shown for comparison (cyt). Scale bars, 5 μm. (TIF) [file pone.0094028.s001.tif]

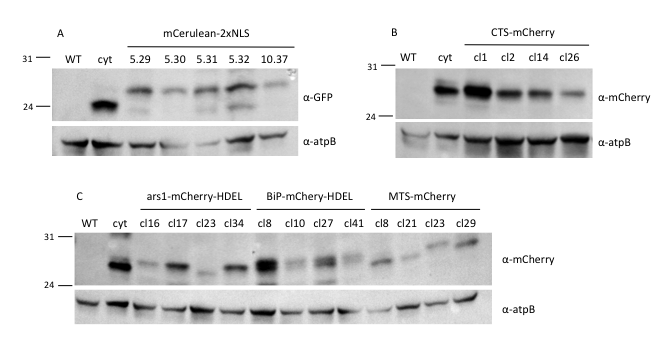

Supplement: Figure S2 — Targeted fluorescent proteins are well-expressed. Immunoblot analysis of total soluble protein from clones transformed with cytoplasmic (cyt) mCerulean (A) or mCherry (B, C) compared to mCerulean targeted to the nucleus (A), mCherry targeted to the chloroplast (B), or mCherry directed to the ER and mitochondria (C). Molecular weight size markers, 31 kDa and 24 kDa, are shown for each gel. Mobility shifts in (A) reflect the addition of the 2xNLS nuclear localization sequence (2.3 kDa). Slight mobility shifts in (B) reflect the addition of the HDEL ER retention sequence (0.5 kDa). Immunoblots probed for atpB are shown as loading controls. (TIF) [file pone.0094028.s002.tif]

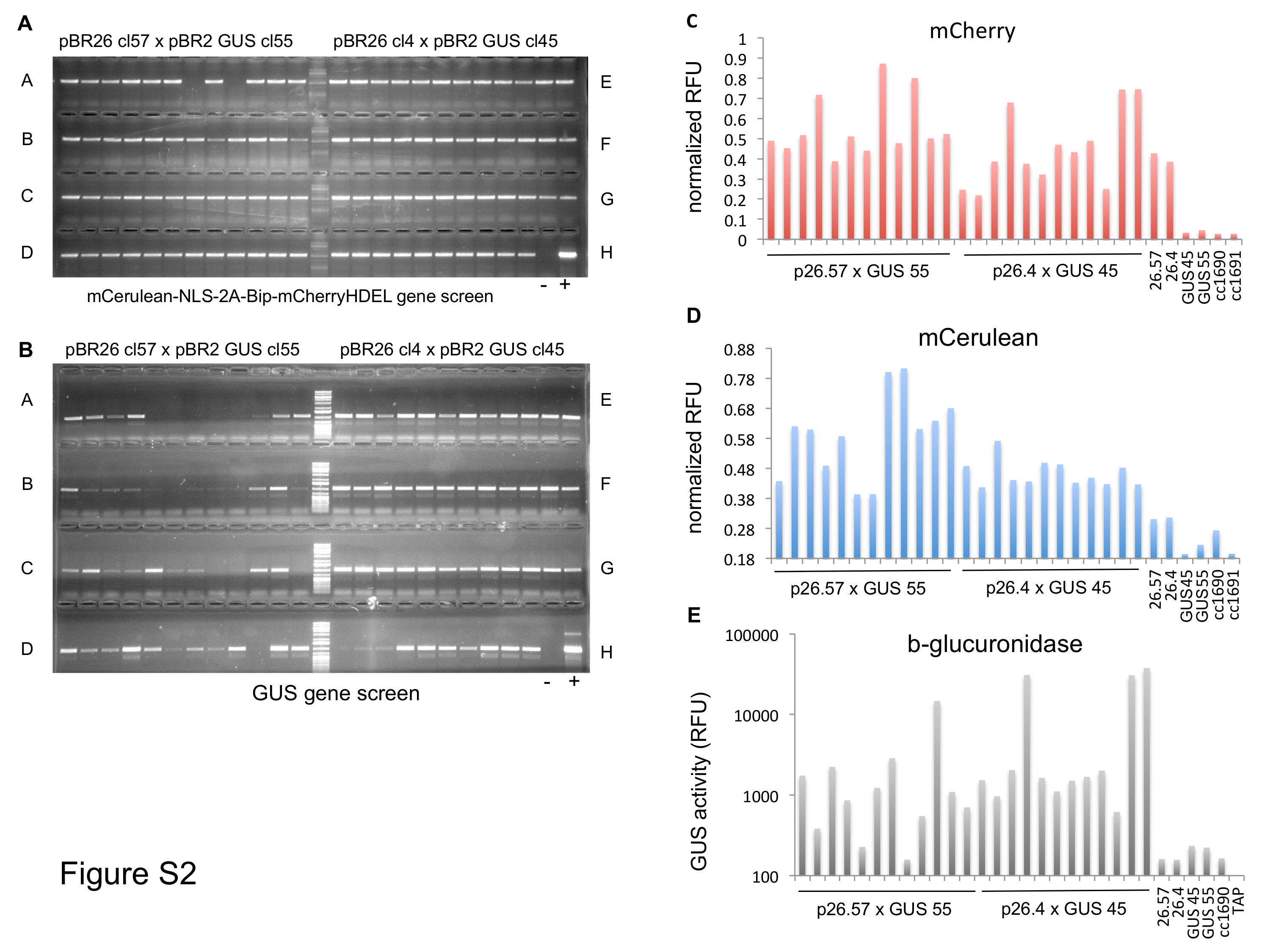

Supplement: Figure S3 — Combining gene-stacking approaches: mating strains transformed with the multicistron vector. Three independent clones stably transformed with pBR26 were mated, pairwise to 3 independent clones expressing β-glucuronidase (GUS). A) A PCR screen with primers shown in Figure 2a on 46 progeny from two representative matings: pBR26 cl57 x pBR2 GUS cl55 (lanes A–D) and pBR26 cl4 x pBR2 GUS cl45 (lanes E–H). (-) cc1690 lysate; (+) pBR26 plasmid. A band indicates the stable inheritance of the multicistron expression cassette from parent to progeny. B) The lysates in (A) were screened with primers specific to GUS. C) Fluorescence microplate reader assay detecting mCherry signals in 12 progeny per mating for the two representative matings shown in a. D) Fluorescence microplate reader assay detecting mCerulean signals in 12 progeny per mating for the two representative matings shown in A. C and D) Y-axis is the ratio of relative fluorescence units of the fluorescent protein to that of chlorophyll fluorescence (ex440/em680). E. Fluorometric GUS activity assay. RFU, relative fluorescence units, reflects the amount of GUS substrate that is catabolized. (TIF) [file pone.0094028.s003.tif]

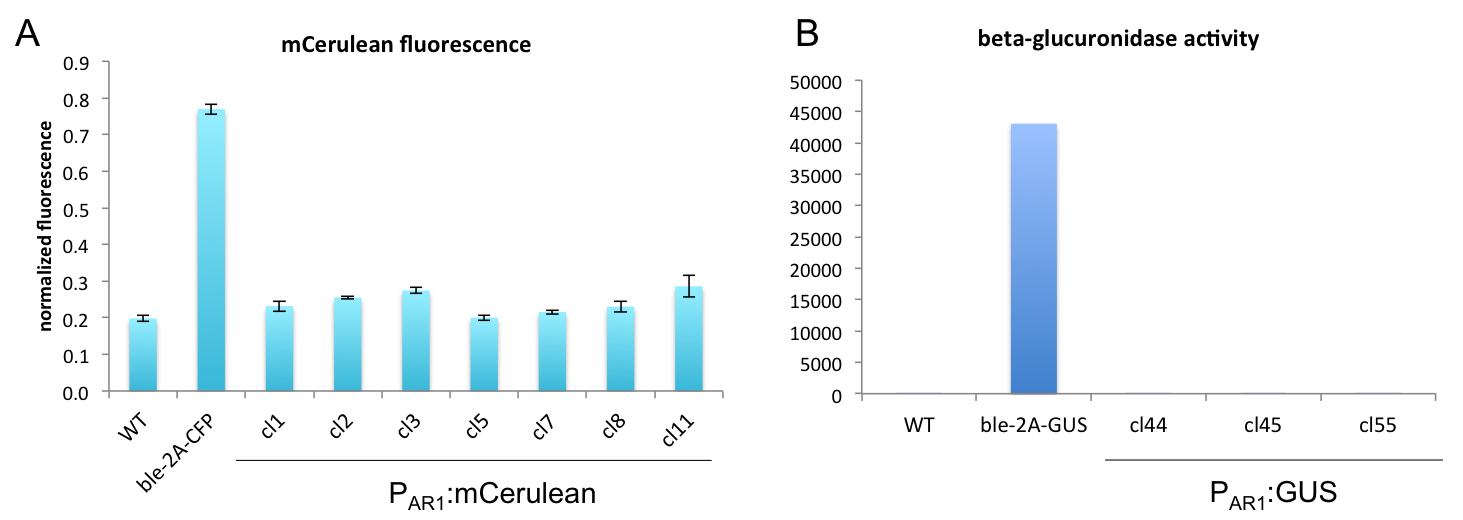

Supplement: Figure S4 — Transgenes expressed from the nuclear genome of C. reinhardtii are subjected to gene silencing. A. Fluorescence plate reader assay on individual transgenic clones expressing PAR1:mCerulean is compared to a clone that is transformed with PAR1:ble2A-mCerulean. The Y-axis represents normalized fluorescence, which is mCerulean fluorescence (ex 450/em486) divided by chlorophyll fluorescence (ex440/em680). B. β-glucuronidase activity assays were performed on individual clones transformed with PAR1:GUS compared to a clone expressing PAR1:ble2A-GUS. GUS activity was monitored by accumulation of fluorescent product. Relative fluorescent units is shown on the Y-axis. (TIF) [file pone.0094028.s004.tif]
